# Supplementary material for: Are carbon emissions trading and green financial instruments synergistic? -Comprehensive quantitative research based on content analysis
Source: PLoS One. 2024 Mar 7;19(3):e0298601. doi: 10.1371/journal.pone.0298601 (PMC10919601; doi:10.1371/journal.pone.0298601)
Supplement: S1 Data — (ZIP) [file pone.0298601.s001.zip › Data/地级市Z_F工作报告.zip!/╔╜╬≈/│ñ╓╬╩╨/└┤╫╘╣1⁄2╓┌║┼í╛┬φ┐╦╩2╛▌═°í┐_2015.docx]

各位代表：

现在，我代表市人民政府向大会报告工作，请予审议，并请市政协委员和其他列席人员提出意见。

2014年工作回顾

过去一年，面对严峻复杂的经济形势，我们认真贯彻落实党中央、国务院和省委、省政府的决策部署，在市委的坚强领导下，全力以赴稳增长、调结构、推改革、促转型、惠民生、强安全、保稳定，各项工作都取得了来之不易的发展进步。

——综合实力持续增强。全市地区生产总值完成1331.2亿元、增长5.1%，固定资产投资完成1245.6亿元、增长14.6%，规模以上工业增加值完成737.2亿元、增长5.1%，公共财政预算收入完成136.3亿元、下降8.3%，社会消费品零售总额完成476.9亿元、增长12%。除财政收入外，其余主要经济指标增速均高于全省平均水平，总体上保持了总量“保三争二”，增速“保六争五”的全省排名。

——产业结构不断优化。粮、经作物种植比由上年的8:2优化为7:3。非煤产业增加值占工业增加值的比重达到32.5%，较上年提高2个百分点。服务业增加值占地区生产总值的比重达到37.3%，较上年提高2.5个百分点。

——就业增收成效显著。新增城镇就业4.34万人，城镇登记失业率为1.8%，低于省控目标。城镇居民人均可支配收入达到24565元、增长8.3%。农村居民人均可支配收入达到10311元、增长11.7%，连续5年快于城镇居民收入增幅。城乡居民收入增幅均高于GDP增幅。

——城市品质稳步提升。长治高新区升级为国家级高新技术开发区，申报成功国家现代农业示范区,长治海关正式批准建立。顺利通过了全国文明城市、国家卫生城市、国家园林城市、全国双拥模范城的复检验收。荣获国家信息消费试点城市、全国优秀民生改善典范城市、全国“六五”普法中期先进市。

一年来，我们迎难而上、应势施策，主要抓了以下七个方面的工作：

一、抓项目、帮企业，经济发展稳中有进

项目建设加速推进。成立了市级项目见效服务办公室，实行“创环境办实事保增长”周报清单制度，办理各类实事4000余件。欣隆、赵庄、高河、华电4个低热值煤发电项目取得“路条”。积极面向长三角、珠三角、京津等地区开展招商工作，引进项目302个、总投资2626亿元。重点实施了942个项目，全年重点项目完成投资1228亿元，总量居全省第二位。有480个项目竣工投产达效，形成了一批新的经济增长点。

扶持企业力度加强。认真落实省政府清理规范涉煤收费政策，全部取消市级和市以下涉煤收费项目，累计为地方煤炭企业减负45亿元，平均吨煤减少近80元。积极解决企业融资难题，召开了三次政银企洽谈会，达成贷款意向343.8亿元。全力扶持民营经济发展，新注册私营企业4490户、注册资本164.6亿元，分别增长86.8%、239.4%；新增小微企业2854户，培育“小升规”企业37户，发展“小巨人”企业18户，全市民营经济增加值增长9.3%。

二、抓转型、调结构，发展方式有了新转变

传统产业提档升级。大力推进现代化矿井建设，11座矿井建成投产，新增产能1200万吨，全市地方煤矿产量达到5663.7万吨、增长4.6%。深入实施煤电互保政策，组织煤炭企业赴河北、山东、河南等地开展促销活动，全市地方煤矿销售5106.2万吨，产销率达到96%以上。加大焦炭企业淘汰整合力度，全市5.5米以上大机焦产能占比由20%提高到55%，焦炉煤气综合利用率达到83%。建立了大用户直供电交易制度，全市3家电厂与2家用电大户签订4.3亿度直供电量，降低企业用电成本近1000万元。

新兴产业不断壮大。围绕七大新兴产业板块，重点实施了198个新兴产业项目，完成投资415亿元、增长16.3%，占工业总投资的71%。现代煤化工板块，大力推进了襄矿40万吨聚氯乙烯、潞宝10万吨己内酰胺、潞安天脊钴基合成油等项目建设，完成增加值21.4亿元、增长11.5%。先进装备制造板块，易通环能低温余热发电成套设备、捷成数控成套设备制造等项目陆续投产，完成增加值36.6亿元、增长15.5%。新能源新材料板块，日盛达光伏玻璃、高科投LED外延芯片及封装、中技金谷新型建材等项目投产发挥效益，完成增加值8.2亿元、增长15.1%。生物医药板块，加快推进了康宝基因工程疫苗、太行中药制剂、邦仕得制药等重点项目，完成增加值8.7亿元、增长0.5%。特色农产品加工板块，林盛果业、潞宝金和生等项目竣工投产，全市农产品加工龙头企业销售收入完成180.1亿元、增长20%，总量居全省第二位。现代物流板块，太行山农产品物流园电子商务平台建成运行，黎城华驰物流园正式运营，全市已运行的较大规模物流园区达到8个，完成增加值112.3亿元、增长7.2%。文化旅游板块，开工建设文化旅游项目42个、完成投资32.7亿元，新增平顺通天峡、襄垣仙堂山2个4A级景区，旅游总收入达到264.7亿元、增长25%。

三、抓创新、促改革，发展活力明显增强

行政审批制度改革扎实推进。全年共取消25项、下放21项、调整7项市级行政审批项目，确认行政许可110项、非行政许可17项。完善了重大项目审批绿色通道，加快重点项目的审批进度。出台了规范行政审批中介服务的意见，对中介机构开展了全面的清理整顿。

土地管理工作进一步规范。出台了市区土地征收储备供应管理办法，对市区土地在规划、征收储备、补偿标准、供应和登记方面实行“五统一”管理。扎实开展了城乡建设用地新机制试点工作，拓展用地空间1万多亩。深入开展违法占地、违法建设专项治理行动，对排查出的“两违”项目进行了分类处置。

金融创新成效明显。成功引入渤海银行、国信证券等金融机构，全市各类金融机构发展到468家。襄垣、壶关2家农信社改制为农商行，潞城农商行首次引入国际先进微贷技术成效显著。大力推广助保贷、信义贷、小微贷等服务中小微企业发展的金融产品，新增各类金融产品100余种，中小微企业新增贷款42.6亿元、增长12.5%，占企业新增贷款的66.6%。第三产业新增贷款42.1亿元，占行业新增贷款的59.8%。全市金融保险业增加值完成68.3亿元，增长5.7%。

科技创新能力得到提升。深化拓展“政校企联合、产学研一体”创新模式，目前已建成产学研一体基地48个。新认定澳瑞特、易通环能等13家国家级高新技术企业，总数达到32家。签订了2.4亿元的技术交易合同。建成国家、省级企业技术中心16个。新认定振东“岩舒”商标为中国驰名商标、总数达到12件。专利授权量739件，发明专利拥有量386件，均名列全省前茅。

四、抓城建、重三农，城乡一体化发展步伐加快

主城区改造建设实施了123项重点工程，“三环八纵十二横”路网框架基本成型，人均城市道路面积达到11.23平方米；同步铺设供热管网30公里、燃气管道41公里、雨污管道113公里，集中供热普及率、燃气普及率、污水处理率分别达到87.1%、92%、92.7%，市政基础设施综合承载能力显著提升。推进实施了581项大县城、重点镇和中心村建设工程，完成投资41.2亿元。全市城镇化率达到48.5%，较上年提高1.6个百分点，增幅全省第二。

现代农业发展步伐加快，新增设施蔬菜10.1万亩，总面积达到46.7万亩，发展速度全省第一。粮食总产量达到16.2亿公斤，实现了“五连增”。新发展农民专业合作社948家、家庭农场302家，累计达到9695家，发展数量连续6年全省第一。“三品一标”认证产品总数达195个。完成17座小型病险水库的除险加固和3条中小河流治理工程，农田水利灌溉面积达到128.3万亩。水土保持治理面积25.5万亩，改造中低产田23.5万亩，主要农作物综合机械化水平达到73%。农村土地承包经营权确权登记颁证试点工作扎实开展。

启动实施了改善农村人居环境“四大工程”，完成投资29.3亿元，21项年度工作任务全面完成。改造农村困难家庭危房15500户，培训新型职业农民10590人，农村“五件实事”年度任务全部完成。扎实开展扶贫开发精准识别工作，在全省率先实现了全市所有贫困重点村定点帮扶全覆盖，完成了2.4万人的易地扶贫搬迁任务，又有4.3万贫困人口稳定脱贫。

五、抓环保、强安全，发展底线进一步强化

持续推进“向污染宣战、享碧水蓝天”的环保攻坚行动，开展了3次“向人民汇报、请人民评议”环保述职评议活动。淘汰落后产能227万吨、取缔黄标车及老旧车10560辆、拆除土小燃煤锅炉1332台。全市万元地区生产总值能耗下降3.6%，6项主要污染物减排指标全部完成省下达目标任务。市区二级以上天数比上年增加53天、达到235天，主要污染物PM10、PM2.5浓度分别下降4.1%和13.9%。完成营造林面积41.2万亩，超额完成省定目标任务34.6个百分点。主城区建成区绿化覆盖率达到45.85%，提高0.53个百分点。

坚持安全生产党政同责、一岗双责、齐抓共管，实行了市县政府班子成员带队突查夜查安全生产工作制度和“两议一报”制度。大力推进安全生产标准化建设，全市所有地方煤矿、161家金属非金属矿山、430家危化企业、161家工贸行业企业，全部达到安全标准化三级以上标准。各类生产安全事故起数和死亡人数分别下降24.9%和1.7%，保持“双下降”态势。亿元GDP死亡率和地方煤矿百万吨死亡率均低于省控目标。

六、抓民生、保稳定，社会事业取得全面进步

公共财政用于民生支出204.3亿元、增长2.9%，占全市公共财政支出的比重达到83.7%。为全市3万名农村义务教育阶段寄宿制学生提供免费营养餐，每人每天餐费标准由0.8元提高到3元，新建、改扩建公办幼儿园18所，改造农村幼儿园35所。深入推进公立医院试点改革，不断巩固“好娃娃”工程成果，全面开展出生缺陷干预救助健苗工程，免费为3.7万名新生儿进行了听力筛查，对1653名新生儿开展了干预救助。积极推进养老、医疗、失业、工伤、生育保险“五险”统征，覆盖率进一步提高，企业养老保险在全省率先实现统收统支。积极开展住房公积金扩面征缴工作，扩面新增44652人。新开工建设各类保障性住房12846套，基本建成11149套，完成投资39.2亿元。

扎实推进“六五”普法，法治长治建设取得新成效。积极排查化解社会矛盾，认真做好信访和人民调解工作。大力实施“六六创安”工程，完善社会治安防控体系，深入开展了打击黑恶势力、打击毒品犯罪等专项行动。集中开展食品药品安全专项整治行动，切实保障了全市人民饮食用药安全。老龄、妇女儿童、残疾人、红十字会、慈善救助等事业健康发展，民族宗教、双拥共建、人民防空、国防动员、民兵预备役等工作取得新成绩，新闻出版、体育健身、文物保护、外事侨务、防震减灾、气象、统计、档案、保密、地方志等各项事业取得新进步。

七、抓廉政、转作风，政府自身建设得到加强

扎实开展第二批党的群众路线教育实践活动和学习讨论落实活动。实行了“市长县长接听”制度，每周由一位市长或县市区长带领相关部门负责同志，在广播电台与广大听众交流互动，解答群众诉求。先后举办了“问农事”、“问安全”、“问民生”、“问作风”4场电视问政活动，解决了一批涉及群众切身利益的问题。严格执行中央“八项规定”和国务院“约法三章”，政府系统会议减少18%，文件精简35%，市本级“三公”经费下降33%。

加强对重点领域、重大投资项目的审计监督，节约政府投资1.4亿元。深化民主法制建设，认真执行市人大及其常委会的各项决议、决定，大力支持人民政协履行职能，积极支持各民主党派、工商联、无党派人士参政议政，全年共办理人大代表建议257件、政协委员提案467件，办复率100%。

各位代表，过去一年，取得的成绩实属不易，这是市委统揽全局、科学决策的结果，是市人大、市政协有效监督、全力支持的结果，是全市各级干部真抓实干、奋力拼搏的结果，是全市人民齐心协力、共同奋斗的结果。在此，我代表市人民政府，向全市人民、驻市部队、武警官兵、公安干警，向各民主党派、工商联、无党派人士和各人民团体，向所有关心支持长治发展的社会各界朋友，表示崇高的敬意和衷心的感谢！

在肯定成绩的同时，我们也清醒地看到，尽管去年我市各项主要经济指标基本保持了稳中有为、稳中有进的态势，但今年以来全国、全省的整体经济形势更加严峻复杂，多重困难和挑战相互交织，经济投资增长乏力，新的消费热点不多，经济稳增长难度更大。一季度全国经济增速进一步放缓，全省工业增加值出现多年未有的负增长。我市经济运行中存在的问题和困难，一是实体经济受影响较大，煤炭需求下降，价格深度下跌，工业生产者出厂价格指数连续35个月低位徘徊；企业经营困难，效益明显下滑，融资难、融资贵问题较为突出。二是多年形成的困扰经济社会发展的深层次问题依然存在，发展规模不大，产业结构单一，“一煤独大”的格局没有根本性改变，传统产业产能过剩问题突出；科技创新能力还不强，民营经济发展还不足；资源环境约束趋紧，大气污染防治形势严峻，节能减排任务艰巨；城乡居民持续增收难度加大，棚户区、城中村改造进展缓慢，安全生产还存在不少薄弱环节。三是政务环境亟待进一步改善，行政审批环节多、办事扯皮效率低，不作为、慢作为、选择性作为、懒政怠政现象仍不同程度存在，“四风”问题还时有发生。对此，我们要直面问题，勇于担当，采取有力措施，认真加以解决。

2015年工作安排

2015年是全面深化改革的关键之年，是全面推进依法治国的开局之年，也是全面完成“十二五”规划的收官之年。今年政府工作的总体要求是：深入贯彻党的十八届三中、四中全会精神，以习近平总书记系列重要讲话精神为根本指针，按照“四个全面”的战略布局，以提高经济发展质量和效益为中心，以改革创新为动力，增强法治意识，保持政治定力，坚持依法行政，全力推进“六大发展”，认真落实“六权治本”，务实推进“五五战略”，推动科技创新、民营经济、金融振兴、扶贫攻坚取得新突破，在帮扶实体经济、推进项目建设、提升园区承载、创优发展环境方面取得新成效，促进社会和谐稳定，奋力开创我市弊革风清、富民强市新局面。

全市经济社会发展的主要预期目标是：地区生产总值增长6.5%左右，固定资产投资增长16.5%左右，公共财政预算收入增长2%左右，社会消费品零售总额增长11%左右，城镇居民人均可支配收入增长8%左右，农村居民人均可支配收入增长9%左右，城镇新增就业岗位4.3万个，城镇登记失业率控制在4%以内，居民消费价格涨幅控制在3％以内。

约束性指标是：万元地区生产总值综合能耗、万元地区生产总值二氧化碳排放量均下降3％，二氧化硫、化学需氧量、氨氮、氮氧化合物、烟尘、工业粉尘减排完成省下达任务，万元工业增加值用水量下降3%。

各位代表，实现以上预期目标决非易事。面对新形势、新常态，我们不仅要看到存在的困难和不利因素，更要看到克服困难、化危为机的利好政策和积极因素。一是国家连续出台了一系列稳增长、调结构、促转型、强改革的政策措施；省委、省政府制定了企业减负60条，将减免全省涉企收费451亿元，可减轻我市企业负担40多亿元，有力地激发企业活力。二是我市一大批国家、省级重点工程今年将开工建设。省政府今年确定十大领域的500多个重点项目有83个在我市，4个低热值煤电厂、4条外送电通道和一大批基础设施、产业发展项目都要开工，大家非常关注的太焦客专今年有望开工建设，重点工程建设要完成投资1000多亿元，将产生巨大的投资拉动效应。三是全市一批优势企业今年将投产达效。去年全市有70个工业项目竣工投产，按计划今年还有58个工业项目竣工投产，还会有20家“小升规”企业进入统计范围，初步预计这些新的经济增长点可实现增加值50亿元以上。四是园区经济的集聚效应今年将进一步显现。去年全市园区实现增加值457亿元，占到全市工业经济总量的62%。今年将重点推进园区的标准化建设，园区经济能够增长7%以上。五是我市城镇化发展空间还很大，每年新增城镇人口5万多人，按照每增加一个城镇人口能拉动10万元投资测算，可带动直接投资50多亿元，是重要的经济增长点。六是消费需求还有很大潜力，随着国家各项鼓励消费政策的落实和新型消费业态的发展，消费预期和消费潜力会进一步增强和释放，对经济增长的贡献会逐步加大。七是大众创业、万众创新的热情空前高涨。去年全市新登记企业4490户，同比增长86.8%，今年一季度新登记企业541户、增长78.6%。随着各项改革措施的深入推进，经济发展活力必将进一步增强。

各位代表，经济发展新常态是新起点、新机遇、新征程、新飞跃，我们必须以新理念、新思路、新作风、新状态，主动学习、主动适应、主动跟进，必须要有坚定的信心和足够的耐心，紧紧抓住提高经济发展质量和效益这个中心，鼓足干劲，抢抓机遇，登高望远，迎难而上，夙夜在公，扎实推进，就一定能够战胜眼前一时的困难，占得即将到来的新一轮发展先机。围绕目标任务，今年我们要重点抓好以下九个方面的工作：

一、切实帮扶实体经济健康发展，着力夯实经济发展基础

实体经济始终是发展的基础，关键在于做实存量。目前，全市有各类实体经济企业1.2万余家，其中煤炭企业124家，规模以上工业企业335家，中小企业2017家，这些企业的产值占到全市经济总量的85%以上，是支撑长治经济发展的基础，是我们当前保持经济增长的关键。要按照做好煤与非煤两篇大文章的思路，切实推动实体经济健康发展。

抓好煤炭企业。按照煤炭产业“六型转变”的要求，今年重点做好以下三方面工作：一是稳定煤炭生产，严格落实煤炭企业清费立税等各项政策措施，保障煤矿正常生产，特别是还没有复工复产的40座矿井要尽快复工复产。今年再竣工投产5座基建矿井，新增产能510万吨，确保全年产量不下降。二是促进煤炭销售，加快煤炭综合信息平台、电子交易平台建设，建立煤炭销售价格协商机制，探索推进煤炭点价交易的期货销售新模式。依托长子能交投物流园、新易达物流园和黎城华驰物流园，建立2个大型煤炭配送中心，与下游煤炭用户形成产业联盟，最大范围辐射周边煤炭市场。力争完成1600万吨以上的公路外销和1450万吨以上的铁路外销，煤炭产销率达到90%以上。三是提高煤炭就地转化率，推动煤炭由燃料向原料转变，实现清洁高效利用。积极推进煤电一体化。抓好以襄垣王桥、潞城店上、郊区西白兔等为主体区域的煤化工园区规划建设，加快推进潞安180万吨煤制油、瑞恒化工60万吨聚氯乙烯项目二期等一批煤化工项目，确保新增煤炭就地转化1800万吨以上，煤炭就地转化率达到50%以上，提高13个百分点。

做优做强非煤重点企业。大力实施“千企百强”工程，特别是要牢牢抓住一批具有举足轻重意义的企业，给予重点帮扶。城区的高科投、中德型材，郊区的首钢长钢、潞安太阳能、澳瑞特；长治县的日盛达、易通环能、振东集团、成功汽车、雅瑞地毯；潞城的天脊、潞宝、华润、卓越；屯留的吉华精细、太行药业、清华钢结构；长子的禾能电厂、浩润食品；壶关的壶化、常平、邦仕得制药、晋通磁材；平顺的大红袍、大唐新能源、文正卓越；黎城的中技金谷、蓝天燃气、晋道酒业；襄垣的山力铂钠、林盛果业、仁达机电；武乡的多维牧业、绿农农牧；沁县的华安焦化、唯思可达、潞宝金和生；沁源的沁新、通洲、天一生态；高新区的玉华再制造、中驰联华、达利食品等等。这些企业是支持长治经济增长的主力军，我们要坚持每月召开一次经济形势分析会，及时预测预警，聘请政府经济顾问，千方百计为企业解决资金短缺、市场开拓、债务纠纷、新产品开发、优势资源整合重组、手续办理等困难和问题，使之开足马力生产，创造更多效益。

积极帮扶小微企业。落实好一系列扶持小微企业发展的优惠政策。财政贴息支持企业开展技术创新活动。实施管理者素质培训、银河培训和专业技能培训，培训3000名中小微企业管理人员，继续支持办好清华大学长治企业家培训班。搭建大中小微企业配套互补合作平台。充分发挥郊区海鸥小企业创业基地的作用，进一步加大对青年创业就业的支持力度。积极推进小微企业创业园区建设。把钜星电子商务产业园建成省级示范基地。进一步深化工商注册登记制度改革，优化登记方式，放松企业经营范围登记管制，推进“三证合一”改革，力争新注册登记企业数量增长15%以上。新培育“小升规”企业20户、“小巨人”企业13户，新创办小微企业2000户。

二、扎实推进项目建设，着力增强经济发展后劲

投资是当前拉动经济增长的第一动力。经济规律告诉我们，越是在经济形势下行时期，投资拉动的关键作用就越明显。

抓好重点领域的投资。今年是全省项目“提质增效年”，做优增量是关键。我们要重点推进十大领域的项目投资，铁路方面重点推进太焦客专等8个项目，完成投资27.5亿元；公路方面重点实施长临、黎左高速公路建设、长邯高速公路改扩建和207、208国道市区过境段改造等15个项目，完成投资37.7亿元；电力及低热值煤发电方面重点推进6个项目，完成投资81亿元；新兴产业方面重点实施376个项目，完成投资465.2亿元；城乡人居环境改善方面实施307个项目，完成投资324亿元。同时，在水利设施、输电通道、煤层气开发利用、节能环保等方面还要抓好205个项目建设，完成投资190亿元。

抓好重大项目的开工建设。目前全市初步确定实施重点工程917项，其中省重点工程83项，市重点工程834项。城区重点推进马坊头商业综合体、博源滨河港湾世贸中心等71个项目开工建设，确保义乌小商品城、烟草物流配送中心等30个项目竣工投产。郊区重点推进漳泽2×100万千瓦改扩建、浪潮云计算中心、中华炎帝城、潞州新世纪商贸综合体、布劳恩电梯等80个项目开工建设，确保潞安太阳能光伏科技大棚、霍家沟7万吨无水乙醇等28个项目竣工投产。长治县重点推进欣隆低热值煤发电、凯德世家商业街区等61个项目开工建设，确保3万吨葡萄酒加工、王庄煤业240万吨洗煤、日益康1万吨主食加工等29个项目竣工投产。潞城重点推进王曲电厂二期、迪威斯促进剂抗氧化等68个项目开工建设，确保潞宝己内酰胺、天正电气、中盛汽车大世界等57个项目竣工投产。屯留重点推进太重榆液长治液压整体搬迁、国药控股医药物流配送中心等53个项目开工建设，确保潞安4万吨高粘度润滑油和2万吨特种环保溶剂油等15个项目竣工投产。长子重点推进高河、赵庄低热值煤发电等28个项目开工建设，确保能交投长子中南铁路集运站、康宝生物雪莲等7个项目竣工投产。壶关重点推进欢乐大世界、大峡谷现代旅游风情小镇等46个项目开工建设，确保中南铁路壶关集运站、新举节能保温材料等28个项目竣工投产。平顺重点推进振东50万亩中药材产业化、虹梯关风景区开发等82个项目开工建设，确保大唐风力发电一期、淜头水电站等60个项目竣工投产。黎城重点推进长邯铁路扩能改造、协鑫30兆瓦光伏发电等65个项目开工建设，确保中技金谷自动化墙板、青春玻璃技改等15个项目竣工投产。襄垣重点推进华电低热值煤发电、中冶集团20万吨取向硅钢、七一能源30万吨聚丙烯等60个项目开工建设，确保恒昌元科技1.5亿只离子电池等7个项目竣工投产。武乡重点推进关河水库供水东干线、红色旅游路二期等59个项目开工建设，确保多维牧业秸秆养羊示范等44个项目竣工投产。沁县重点推进中老年营养小米粉、3万吨有机肥等48个项目开工建设，确保华安焦化6.8万吨煤气制天然气、沁园春矿泉水等39个项目竣工投产。沁源重点推进晖源20万吨石油压裂支撑剂二期、沁新氢氧化铝、通洲144万吨焦化等66个项目开工建设，确保太岳山风电二期、联鸿20兆瓦光伏发电等33个项目竣工投产。高新区重点推进炎黄照明LED灯芯、汇灵科技印刷纸质电子标签等29个项目开工建设，确保康宝基因疫苗、奥盛工程机械再制造、世龙食品改扩建等8个项目竣工投产。这些项目是保证长治今后可持续发展的动力所在，我们必须夜以继日，只争朝夕，加快推进开工建设，必须将项目开工建设投产按月分解任务，倒排项目工期，责任到人，加大追责问责力度，确保完成任务。

抓好新项目的签约储备。抢抓“一带一路”、京津冀一体化、综改区建设等国家战略机遇，主动融入、积极对接。按照全省部署赴北京、广州、上海、武汉等地开展招商推介，引进一批既符合国家产业政策，又与我市产业发展相关联的真转型、高科技、有前景的项目，力争新签约项目投资总额完成1800亿元。特别要注重产学研对接，每个县市区至少对接1至2家国内外一流高等院校、科研院所，建立起长期有效的联系合作机制，形成创新驱动的新引擎。

三、大力推进标准化集约化园区建设，着力打造新的经济增长极

目前全市有各类工业园区27个，其中国家级示范园区1个，省级示范园区4个，入园企业共411家，主要涉及现代煤化工、先进装备制造、LED光电、新能源新材料、医药制造等产业。2014年园区经济占全市GDP的34.3%，园区已成为全市经济的主要承载。但我们的园区建设普遍存在建设标准不高、基础设施不完善、集约化服务不到位、土地投入产出率低的问题。进一步提升园区综合承载能力迫在眉睫。

创新园区发展模式。建立“一区多园”发展模式，将郊区漳泽工业园、城区城南机械工业园、长治县科工贸产业园、屯留康庄工业园和煤化工业园、潞城翟店工业园、襄垣王桥和富阳工业园等8个园区作为高新区的辐射区，实现资源共享、优势互补，通过飞地经济、效益共享等模式，实现高新区拓宽发展空间、其它园区得到经济效益的双赢局面。

优化园区空间布局。根据地域分布、产业关联度等因素，对全市现有园区进行整合，最大程度发挥园区优势，避免“一企一园”发展模式。将郊区南耀、霍家工业园整合为寒山工业园，潞城潞宝、史回、天脊工业园整合为潞城煤化工业园，逐步推进沁源沁绵、太岳工业园整合。推动园区提档升级，城区城南机械工业园要积极创建国家级军民融合产业示范基地，襄垣王桥工业园要加快创建国家级煤化工产业示范基地，长治县科工贸产业园、襄垣富阳工业园要全力创建省级工业新型化产业示范基地，确保全市省级以上工业示范基地总数达到7个以上。

提升园区发展效益。各类园区要设定基本投入产出门槛，根据产业类型、投资强度、项目预期效益等对入园项目进行评估，真正引进一些符合园区产业定位，发展前景好的项目。今年要大力开展园区项目全面清理整顿工作，对那些占地多年、没有发挥效益的企业要坚决依法清理、优存劣汰，切实解决园区土地闲置和低效利用的问题。今后新上项目原则上要全部进园区。

健全完善园区功能。目前的园区有一半左右都是2010年以后成立的，园区基础设施不完善、功能不全，有的甚至还没有实现“五通一平”。要按照“先地下后地上”的原则，统筹推进园区水、电、暖、路、气、通讯、物流、垃圾和污水处理等基础设施建设，发挥园区综合效益。同步完善商务、医疗、休闲等生活设施，为企业家创业兴业和职工工作生活提供一个良好的环境。进一步健全园区管理机构，提升服务水平，对进园区项目给予“保姆式”、“代办式”服务，帮助企业解决后顾之忧。

四、坚定不移改革创新，着力优化发展环境

大力改善政务环境。重点做好两个方面的工作。一是优化便民服务，将所有行政审批事项、公共资源交易事项、与群众生活密切相关的公共服务事项全部纳入政务服务中心，实行首席代表负责制、服务承诺制和限时办结制，切实做到项目审批进中心，中心之外无审批，真正实现“一厅式”办公和“一条龙”服务，让群众“进一个门、办所有事”，切实解决“办证难”的问题。二是加速项目审批，进一步优化审批流程，压缩项目审批时限。特别要进一步完善至少一月两次的项目联审联批制度，进一步大力整治“红顶中介”，破除中介服务垄断，清理中介服务事项，规范中介服务收费和时限，切实加快行政审批速度，去除行政审批负担，优化行政审批服务。充分发挥项目见效服务办公室的作用，畅通问题收集渠道，创新问题解决机制，及时解决项目建设中存在的问题。

加强土地资源管理。加快土地利用总体规划的调整，确保今年上半年完成不涉及基本农田的规划调整。坚守耕地红线，确保541万亩耕地总量动态平衡、443万亩基本农田不减少。用好节约集约用地考核等四项用地新机制，有效破解企业用地瓶颈。严格落实市区土地“五统一”管理，坚决打击违法占地行为，促进土地市场走上规范有序的轨道，力争全年土地收益达到15亿元。

推进金融创新发展。积极引进各类银行、证券等金融机构，力争全市各类金融机构达到500家。建立PPP项目支持基金、众筹引导基金、应急周转基金和市级担保联盟，构建新型融资模式。继续发展以“一区一会三平台”为核心的金融服务体系，加快金融商务区建设，完善政银企联席会议制度，推动长治民间融资服务平台、股权托管平台、金融信息系统进入实质性运作。加快康宝、成功、潞安太阳能等企业上市步伐，力争壶化集团年内上市，支持襄子老粗布、永腾建材、澳瑞特等优势企业在新三板、天交所、山西股权交易市场等交易平台挂牌交易。大力推广潞城农商行无抵押无担保小额信贷的经验和做法。积极推进县级农信社改制。创新发展互联网金融，加快推进郊区环渤海金融电子商务基地建设。进一步完善诚信体系，探索建立应收账款债权流转平台，防范和打击非法集资，优化金融生态环境。力争全市金融保险行业增加值完成70亿元，占GDP的比重达到5%。

积极拓宽财政财源渠道。完善政府预算体系，按照保民生、保基本的原则，加强财政资金使用绩效管理，强化“过紧日子”的思想，花好财政的每一分钱。千方百计运用多种融资方式，加大支持公益性项目建设力度，精打细算用好25.9亿元的政府债券；整合市级国有参控股公司的资产，进一步充实长治市投资建设开发公司的净资产，力争申报发行15亿元的城投债券；积极推广政府与社会资本合作PPP模式，公布向社会资本开放的建设项目名单。

全力抓好科技创新。加快长治技术交易市场建设，确保年内开通运行。搭建创新公共服务平台，实现科技成果转化、创业合作、技术供求等信息及仪器设备互通共享。积极组织企业走出去，主动与“科研高地”对接，开展2次以上技术项目对接会，确保全年技术交易额完成3亿元以上。培育造就一批有影响、创新能力强的人才队伍。选树一批成长性好的科技创新项目和企业予以重点扶持，在技术认证、项目示范、产品推广、品牌创建等方面强化跟进服务。充分发挥长治国家级高新技术开发区的引领示范作用，加快创新企业孵化基地建设，新创建1个国家级技术中心、2个省级技术中心、10个市级技术中心，新培育6家国家级高新技术企业，确保全年新技术、新工艺、新装备对工业增加值的贡献率达到41%以上。

五、全力以赴做好“三农”工作，着力打牢农民持续增收的基础

加快发展现代农业。围绕全国优质玉米示范区和北方设施蔬菜样板区两大主题，加快推进国家现代农业示范区建设，建设20个千亩玉米高产示范片区、30个高标准现代设施农业高效示范园区，新培育玉米制种5千亩，新发展设施蔬菜10万亩，加快建立新技术、新品种、新机具试验示范基地，主要农作物综合机械化水平再提高1个百分点。逐步完善农田水利、田间道路等基础配套设施建设。支持培育专业大户、家庭农场、农民合作社等新型经营主体。大力扶持农业产业化龙头企业做强做大，力争全市农产品加工龙头企业销售收入突破190亿元。

多渠道促进农民增收。认真落实各项强农惠农富农政策，确保各类惠农补贴资金及时足额发放到位，提高农民转移性收入。新发展4.5万亩小杂粮、5万亩干果经济林、15万亩生态经济型灌木林、15万亩特色中药材、1千亩食用菌、60个高产果园，新建改扩建100个标准化规模养殖场，不断提高农民经营性收入。继续推广公司+基地+农户的产业模式，促进农村劳动力就近就地转移就业，努力提高农民工资性收入。持续增加农民财产性收入，确保农民收入增幅高于城镇居民。

不断深化农村改革。全面开展农村土地承包经营权确权登记颁证工作，6月底前完成全市辖区航拍任务并将航拍工作底图分发各县市区，确保今明两年完成全市的土地确权登记颁证。建立完善农村土地流转交易市场，有序推进农村土地经营权流转，引导发展农业适度规模经营，建立健全农村土地流转服务体系和纠纷调解仲裁体系。

扎实开展扶贫攻坚。实施好产业扶贫，抓好国家和省财政专项资金扶持的62个整村推进产业扶贫项目和5个乡村旅游扶贫项目。实施好金融扶贫，通过贷款贴息政策，支持扶贫企业和贫困户发展。实施好科教扶贫，对1万名以上贫困地区农村劳动力开展技能培训，对考入二本以上大学的贫困生，每人一次性资助5000元，阻断贫困代际传递。实施好精准扶贫，完善党员干部驻村帮扶机制，实现机关单位党员干部精准帮扶贫困县贫困户全覆盖。确保再有4.84万贫困人口稳定脱贫。

六、积极稳妥推动新型城镇化进程，着力提升城乡居民生活质量

加速推进主城区提质建设。巩固“大干城建年”成果，重点实施125项城建工程。新建改造12条市政道路、6座市政桥梁、3座人行过街天桥、3座铁路立交桥、1座互通立交桥、20条背街小巷，实施城市公共自行车服务项目，加快建设智慧交通体系。重点推进21个城中村改造、7个棚户区改造项目。大力实施供水、供气、供热、排水、照明、绿化、污水处理、垃圾处理等市政基础工程，加快推进“三河一渠”综合治理工程。全市新开工各类保障性住房15792套，基本建成14316套。同时，要以智慧城市建设为龙头，依托“互联网+”，切实抓好服务业发展，统筹推进电子商务、现代物流、信息服务等生产性服务业和大众消费、旅游休闲、健康养老等生活性服务业。

积极推进上党城镇群建设。以潞城市经济社会发展规划、城乡发展规划和土地利用规划“三规合一”试点工作为引领，进一步完善上党城镇群建设规划。充分发挥6条城际连接线的综合效应，加快“内圈”6个卫星城与主城区的设施对接、产业对接、机制对接、服务对接，加强互联互通，实现资源共享。着力提升“外圈”5个大县城对人口和产业的承载力。加快推进22个重点镇和42个中心村建设。深化户籍制度改革，全面放开建制镇和县城落户限制，逐步将城中村、城郊村和产业聚集区内村庄的农业人口成建制地转为城镇人口，今年力争完成6万人左右的落户任务，全市城镇化率再提高1.7个百分点左右。

大力实施农村人居环境改善“四大工程”。完善提质工程重点是改建500公里村通水泥 （油）路，改善和提高7.85万农村人口的饮水安全标准，建设50个生态村庄，新建50个农村老年人日间照料中心，完成58793户广播电视卫星户户通任务。农民安居工程重点是完成7313户的采煤沉陷区治理搬迁、322户的地质灾害搬迁工作。环境整治工程重点是完成27个村的污水防治试点工作，全面开展农村生活垃圾专项治理。宜居示范工程重点是抓好23个省级、40个市级、295个县级美丽宜居示范村建设。扎实办好农村 “五件实事”，再改造农村困难家庭危房7787户，新建改扩建农村幼儿园35所，异地搬迁农村贫困人口9752人，深入推进乡村清洁工程，培训新型职业农民1万人。

七、持之以恒改善生态环境，着力建设生态文明

全面完成“十二五”节能减排目标。在节能方面，积极推进国家新能源示范城市建设，大力培育和发展节能产业，推进十大节能工程，实施74个节能技改项目，确保全年节约标煤130万吨以上。在减排方面，加快市污水处理厂二期工程进水管网和脱泥设备的安装，尽快拆除长源焦化、市煤气公司气源厂、犇盛化工的关停生产设施，建成73个规模化养殖场的畜禽粪污处理设施。

突出抓好水、大气、土壤三个治理重点。水污染防治要启动建立从水源保护到安全供水的全过程监管机制，科学防治地下水污染，确保饮用水安全。严格控制入河排污总量，重点推进沁河、浊漳河流域水环境综合治理。严格实行跨界断面水质考核。完成主城区应急备用水源工程建设。加强污水处理日常监管，确保所有城镇生活污水处理厂稳定达到一级排放标准。重点建制镇年内全部建成污水处理设施。大气污染治理要积极推进主城区燃煤锅炉清洁能源的替代工作，淘汰燃煤锅炉467台。加快漳山电厂、王曲电厂供热改造。推进24家焦化、5家电力、5家钢铁、5家水泥等重污染企业的对标升级改造。加快落实油品升级，继续淘汰黄标车、老旧车，推广清洁能源车应用。确保PM10、PM2.5浓度分别下降4%和8%以上。土壤污染防治要紧紧抓住农用地和建设用地两个重点，明确保护、禁止、限制和控制的措施要求，组织开展土壤环境例行监测。探索划定土壤环境优先保护区域并落实保护责任。

创新环境保护机制。严格执行新的环境保护法，建立完善环保部门与公安机关联合执法的工作机制，深入开展全市环境保护大检查。加大对违法企业的查处力度，对排污企业采取按日计罚、查封扣押、停产限产、行政拘留等最严厉的处罚措施。依法公开环境信息，完善公众参与机制，环境违法信息记入社会诚信档案，及时公布违法者名单。大力推进环境污染第三方治理，按照政府引导、市场化运作、排污者付全费的原则，对污染物实施专业化治理。加强工作考核和责任追究力度，加快构建多部门共同负责的环境保护工作新体系。

大力推进造林绿化和生态修复。今年重点做好两项工作。一是加大造林绿化力度，积极推进林业“六大工程”建设，完成营造林40万亩，力争森林蓄积量增加100万立方米，森林覆盖率提高一个百分点。二是突出抓好采空区、沉陷区治理，启动7个县市区的18个乡镇、46个村、2.4万人的采煤沉陷区治理工程。

八、统筹协调各项社会事业发展，着力保持社会和谐稳定

推进教育事业发展。优化整合职业教育资源，加快长治职教园区建设。启动普通高中办学标准化建设，优化学校布局，改善教学软硬件设施，切实提高普通高中教育质量。全面改善义务教育薄弱学校和寄宿制学校基本办学条件。推进义务教育办学模式改革，继续实施“联盟校”办学模式，实现优质教学资源共享。再新建、改扩建18所城镇公办标准化幼儿园，完成主城区3所公办幼儿园建设。认真落实特殊教育提升计划。加大城乡教师交流、校长轮岗力度，交流比例不低于10%。

大力促进就业创业。把稳定和扩大就业作为经济运行合理区间的下限。延缓困难企业缴纳社会保险费，给予社会保险、就业岗位和职业培训等补贴，确保困难企业职工不失业。大力发展服务业和小微企业，增加就业机会。提供更多的公益性岗位，保障就业困难群体实现就业。深入开展创业型城市建设，鼓励大众创业、万众创新，落实好国家给予小微企业一次性创业就业补助每人1000元、经营场地租金补贴每年2000元的政策。认真落实大学生创业“七补一贷”政策，加快大学生创业实训基地建设，每个县市区至少要建立1个创业孵化基地或创业园。

不断提升医疗卫生水平。巩固和发展县级公立医院改革成果，实现全市县级公立医院改革全覆盖。加大优质医疗资源下沉力度，抓实抓好“进村接地气、入户送健康”活动。完善城乡居民大病保险制度。为农村60岁以上老人实行免费体检。优质高效推动城乡基本公共卫生服务均等化。做好突发事件卫生应急处置工作。加快长治市传染病医院、精神卫生中心等专科医院建设。深入实施出生缺陷干预救助健苗工程。切实做好人口计生工作。

健全完善社会保障体系。今年在财力十分困难的情况下，继续保持财政对社会基本保障投入的稳定增长，进一步提高企业退休人员基本养老金、城乡居民基础养老金、城乡低保、农村五保对象补助水平。建立工资正常调整和增长机制，确保机关事业单位养老保险制度改革、公车改革和乡镇工作补贴落实到位。积极推进社会保险扩面征缴工作，继续扩大“五险”统征范围。住房公积金新增扩面3.5万人。开工建设市级救灾物资储备库。全面开展临时救助工作，让遇到急难特困的群众求助有门、受助及时。

大力发展文化事业。加快推进市直四大文化院团排练场建设。组织开展好第二十五届全国书博会长治分会场活动。确保市新闻大楼在6月底前全面入驻，切实提升广播电视节目的制作水平和播出质量。大力传承红色基因，不断加强廉政文化建设。切实加大长治旅游品牌宣传推介力度。积极申办第十五届省运会。强化文物、传统村落和非物质文化遗产保护。举办好中国人民抗日战争暨世界反法西斯战争胜利70周年纪念活动。

积极创新社会治理。全面推进依法治市，加大法治长治建设力度。扎实做好信访工作，采取有力措施解决好信访案件，有效预防和化解社会矛盾。加强社会治安综合治理，以更大的力度严厉打击黑恶势力、毒品犯罪、“两抢一盗”和治安乱点。积极推进“天眼工程”建设。筹建长治市自愿戒毒专科医院。切实加强应急管理和网络舆情工作，有效应对公共突发事件。依法管理民族宗教事务。创新社区管理机制，实现网格化管理全覆盖。开展村务公开专项整治，提升基层民主管理服务水平。

全面开展城乡爱国卫生清洁运动。大力推进食品安全城市创建，加快食品检测中心建设。做好气象、地震、档案、保密、国防教育、人民防空、民兵预备役、双拥共建、外事侨务、对台、地方志等工作，发展老龄、妇女儿童、残疾人、红十字会等事业。

九、毫不松懈抓好安全生产，着力提高安全发展水平

当前，经济下行压力持续加大，企业效益下滑，高危企业的亏损增加。受此影响，一些企业把精力更多地放在了融资、销售和扭亏上，对安全生产的关注度下降了，抓的力度减弱了，应该花在保安全生产上的经费减少了。同时，一些企业有能力的管理和工程技术人员“跳槽”增多，员工流动性加大。因此种种，我们面临的安全形势十分严峻，安全生产不容乐观。今年我市有一座煤矿，在不到一个月的时间内连续发生两起安全生产责任事故，共造成3人死亡。血的沉痛教训再次警醒我们，越是在经济困难的时期，越要高度重视安全生产工作。各级政府监管部门要严格安全监管执法，以最坚决的态度坚守生命红线，以最严格的措施落实安全生产各项法律法规和规章制度。所有企业要抓紧抓实抓好安全生产，在思想上时刻清醒，在行动上必须坚实。企业效益再下滑，但安全管理千万不能下滑，企业收益再减少，但必须的安全投入不能减少。制度落实要严之又严，隐患排查要细之又细，现场管理要实之又实；杜绝整改隐患优柔寡断、设备更新长期拖延、安全培训“偷工减料”，坚决落实新的安全生产法，以铁的制度、钢的落实，坚决遏制重特大事故，防范较大事故，减少一般性事故。

各位代表，新形势下加强政府自身建设的任务更加突出。一要依法行政，六权治本。按照依法确定权力、科学配置权力、制度限制权力、阳光行使权力、合力监督权力、严惩滥用权力的要求，以行政管理层级、行政职权主体为重点，进一步厘清权力边界，明确责任主体，完善制度体系，实施流程再造，有序公布权力清单、责任清单、负面清单，严格监控权力运行的“关键点、缺失点、风险点”。市级政府部门要在年底前编制完成权力清单，已确定的两个试点县区和15个试点单位年底前正式启动实施“六权治本”。严格执行“三重一大”制度。自觉接受人大及其常委会的监督，主动接受政协的民主监督，认真办理人大代表建议和政协委员提案，广泛听取民主党派、工商联、无党派人士和人民团体意见。二要廉洁从政，严惩腐败。各级政府党组要切实履行党风廉政建设主体责任，强化“一岗双责”。严格落实中央“八项规定”、国务院“约法三章”和省市各项规定，严控“三公”经费支出。围绕煤焦、土地、交通、房地产等重点领域和资源配置、工程招标、政府采购、资金分配等关键环节，扎实开展专项整治，切实形成并保持惩治腐败的高压态势。三要务实勤政，改进作风。巩固党的群众路线教育实践活动和学习讨论落实活动成果，扎实开展“三严三实”专题教育活动。突出问题导向，抓好整改落实，持续狠刹“四风”。充分发挥监察、审计部门的监督作用。加大督办考核力度，确保工作任务落到实处。从严管理公务员队伍，把纪律挺在前面，严厉惩处失职渎职，坚决整治慵政懒政怠政行为，切实解决变相的吃拿卡要和门好进、脸好看、事依然难办等顽症。民之所望，施政所向。在长治发展的关键时刻，政府的每一名工作人员都要牢固树立勤政为民的理念，始终保持奋发有为的精神状态，兢兢业业、无私奉献，言必行、行必果，拿出苦干实干拼命干的劲头抓落实，不出成果不松手，不达目标不罢休，切实用好人民赋予的权力，创造出无愧于人民的业绩。

各位代表，潮起海天阔，扬帆正当时。让我们紧密团结在以习近平同志为总书记的党中央周围，在省委、省政府和市委的坚强领导下，紧紧依靠全市人民，攻坚克难，开拓创新，为全面落实“六大发展”，务实推进“五五战略”，奋力开创弊革风清、富民强市新局面而努力奋斗！
